# Preoperative diagnosis and prediction of hepatocellular carcinoma: Radiomics analysis based on multi-modal ultrasound images

**DOI:** 10.1186/s12885-018-5003-4

**Published:** 2018-11-12

**Authors:** Zhao Yao, Yi Dong, Guoqing Wu, Qi Zhang, Daohui Yang, Jin-Hua Yu, Wen-Ping Wang

**Affiliations:** 1 Department of Electronic Engineering, Fudan University, No. 220, Handan Road, Yangpu District, Shanghai, 200433 China; 2 Department of Ultrasound, Zhongshan Hospital, Fudan University, 180 Fenglin Road, Shanghai, 200032 China

**Keywords:** Shear wave dispersion, Viscoelasticity, Radiomics approach, Ultrasound, Hepatocellular carcinoma

## Abstract

**Background:**

This study aims to establish a radiomics analysis system for the diagnosis and clinical behaviour prediction of hepatocellular carcinoma (HCC) based on multi-parametric ultrasound imaging.

**Methods:**

A total of 177 patients with focal liver lesions (FLLs) were included in the study. Every patient underwent multi-modal ultrasound examination, including B-mode ultrasound (BMUS), shear wave elastography (SWE), and shear wave viscosity (SWV) imaging. The radiomics analysis system was built on sparse representation theory (SRT) and support vector machine (SVM) for asymmetric data. Through the sparse regulation from the SRT, the proposed radiomics system can effectively avoid over-fitting issues that occur in regular radiomics analysis. The purpose of the proposed system includes differential diagnosis between benign and malignant FLLs, pathologic diagnosis of HCC, and clinical prognostic prediction. Three biomarkers, including programmed cell death protein 1 (PD-1), antigen Ki-67 (Ki-67) and microvascular invasion (MVI), were included and analysed. We calculated the accuracy (ACC), sensitivity (SENS), specificity (SPEC) and area under the receiver operating characteristic curve (AUC) to evaluate the performance of the radiomics models.

**Results:**

A total of 2560 features were extracted from the multi-modal ultrasound images for each patient. Five radiomics models were built, and leave-one-out cross-validation (LOOCV) was used to evaluate the models. In LOOCV, the AUC was 0.94 for benign and malignant classification (95% confidence interval [CI]: 0.88 to 0.98), 0.97 for malignant subtyping (95% CI: 0.93 to 0.99), 0.97 for PD-1 prediction (95% CI: 0.89 to 0.98), 0.94 for Ki-67 prediction (95% CI: 0.87 to 0.97), and 0.98 for MVI prediction (95% CI: 0.93 to 0.99). The performance of each model improved when the viscosity modality was included.

**Conclusions:**

Radiomics analysis based on multi-modal ultrasound images could aid in comprehensive liver tumor evaluations, including diagnosis, differential diagnosis, and clinical prognosis.

## Background

Hepatocellular carcinoma (HCC) is the most common type of primary liver cancer and the most common cause of death in people with liver cirrhosis [1]. Early and accurate diagnosis of HCC is of vital importance in clinical decision-making and treatment. Currently, although various treatments have been proven effective in the treatment of HCC, recurrence remains an important clinical challenge, with its aggressive biological behaviour and negative impact on overall patient survival. Conventional B-mode ultrasound (BMUS), as a non-invasive, easy and safe procedure, is currently the first-line imaging modality for the diagnosis of HCC. However, BMUS has a limited role in the clinical diagnosis of focal liver lesions (FLLs) and of complicated recurrent lesions. Recent technical advances in shear wave elastography (SWE) and viscosity ultrasound increase the diagnostic efficiency of ultrasound and allow it to evaluate liver stiffness with the aim of assessing hepatic fibrosis and cirrhosis. To date, only a few studies have focused on the quantification of SWE stiffness in FLLs [2–4].

More recently, as an emerging method for medical image processing, radiomics is used to convert medical images into high-dimensional, mineable features that reflect underlying pathophysiological information [5]. Radiomics employs a variety of state-of-the-art machine learning or deep learning techniques to complete a variety of clinical tasks, which greatly pushed the development of precision medicine [6]. Microvascular invasion (MVI) and antigen Ki-67 (Ki-67) are regarded as high-risk factors for HCC recurrence. As an immunotherapy target, programmed cell death protein 1 (PD-1) has also become increasingly meaningful for the treatment of patients with HCC.

According to previous research, radiomics has great potential for the diagnosis and treatment of liver diseases. In a study by Virmani et al. [7], 48 features were extracted from gray-scale ultrasound images to differentiate normal livers, cirrhotic livers and HCC. A genetic algorithm and support vector machine (SVM) were used as feature selection and classification methods. Owjimehr et al. [8] performed a wavelet packet transform on the gray-scale ultrasound image and extracted 61 features to differentiate normal, fatty and heterogeneous livers. SVM and k-nearest neighbour classifiers were applied to classify the images into three groups. Furthermore, some studies applied artificial neural networks to diagnose abnormal livers [9], chronic liver disease [10] and HCC malignancy [11]. These studies demonstrate the feasibility of ultrasound imaging in liver disease diagnosis and imply the great potential of radiomics analysis.

Current radiomics methods have several limitations when analysing the data of our study. First, traditional engineered features (intensity, shape, margin, calcification, wavelet, etc.) are designed for different diseases and are poorly adaptable for HCC. Second, the deep learning algorithms are easily over-fitted when dealing with data with a small sample size. Finally, most of the above studies used a single modal ultrasound imaging, without utilizing comprehensive information provided by multi-modal ultrasound images.

Due to its good performance in signal representation and reconstruction, sparse representation (SR) is widely used in feature selection [12, 13] and image classification [14, 15]. By training the optimal texture to represent images, SR can adaptively learn image features with a small amount of imaging data. Furthermore, as a nonparametric model, SR can effectively avoid over-fitting and has strong robustness [16].

A radiomics analysis system based on SR and SVM was proposed in our study. We trained the model with multi-modal ultrasound images and used histology as the gold standard measure. Our study aims to evaluate the feasibility of ultrasound radiomics models in the differential diagnosis and characterization of histologically proven FLLs and to determine an initial prognosis of HCC.

## Methods

### Patients and materials

Between July 2017 and June 2018, 177 consecutive patients (102 women and 75 men; age range: 15–91 years, mean: 55.5 ± 10.4 years) who were referred to our institution for FLL SWE assessment were included in the prospective study. For patients under the age of 16 years, informed consent was obtained from a parent and/or legal guardian. All patients underwent multi-modal ultrasound examination, including BMUS, shear wave elastography (SWE), and shear wave viscosity (SWV) imaging before surgery. The final diagnoses for all 177 patients were based on histopathological results obtained from liver biopsy during surgery.

Data collected included the patient’s age, gender, and focal liver lesion location. Among all 177 patients included in this study, 66 were excluded, and the exclusion criteria were as follows: (1) missing important pathology results; (2) poor imaging quality; (3) accompanied with other diseases, including cirrhosis and fatty liver. Characteristics of the 111 FLL patients enrolled are summarized in Table 1, which include patient gender and age. Statistics show that the gender and age of patients are related to the benign and malignant classification of tumors (*p* < 0.05). In addition, patient age was statistically related to the type of malignant tumor (p < 0.05).

**Table 1:** Beseline characters of patients

Parameters	All patients	Male (N; %)	Ages (mean ± variance)
Tumor category			
benign	46	21; 46%	50.5 ± 13.4
malignant	65	54; 83%	56.6 ± 8.3
P value	**–**	0.00004	0.0040
Malignant subtyping			
HCC	47	41; 87%	55.3 ± 8.4
others	18	13; 72%	60.2 ± 7.0
P value	**–**	0.1550	0.0267
PD-1 prediction			
PD-1 present	15	14; 93%	53.0 ± 8.8
PD-1 absent	24	20; 83%	56.2 ± 8.9
P value	**–**	0.3831	0.2782
Ki-67 prediction			
≤ 25	21	19; 90%	53.9 ± 9.6
> 25	23	19; 83%	56.6 ± 7.6
P value	**–**	0.4647	0.2441
MVI prediction			
MVI present	21	18; 86%	53.9 ± 8.0
MVI absent	22	19; 86%	56.0 ± 8.9
P value	**–**	0.9677	0.3810

**Fig. 1:**
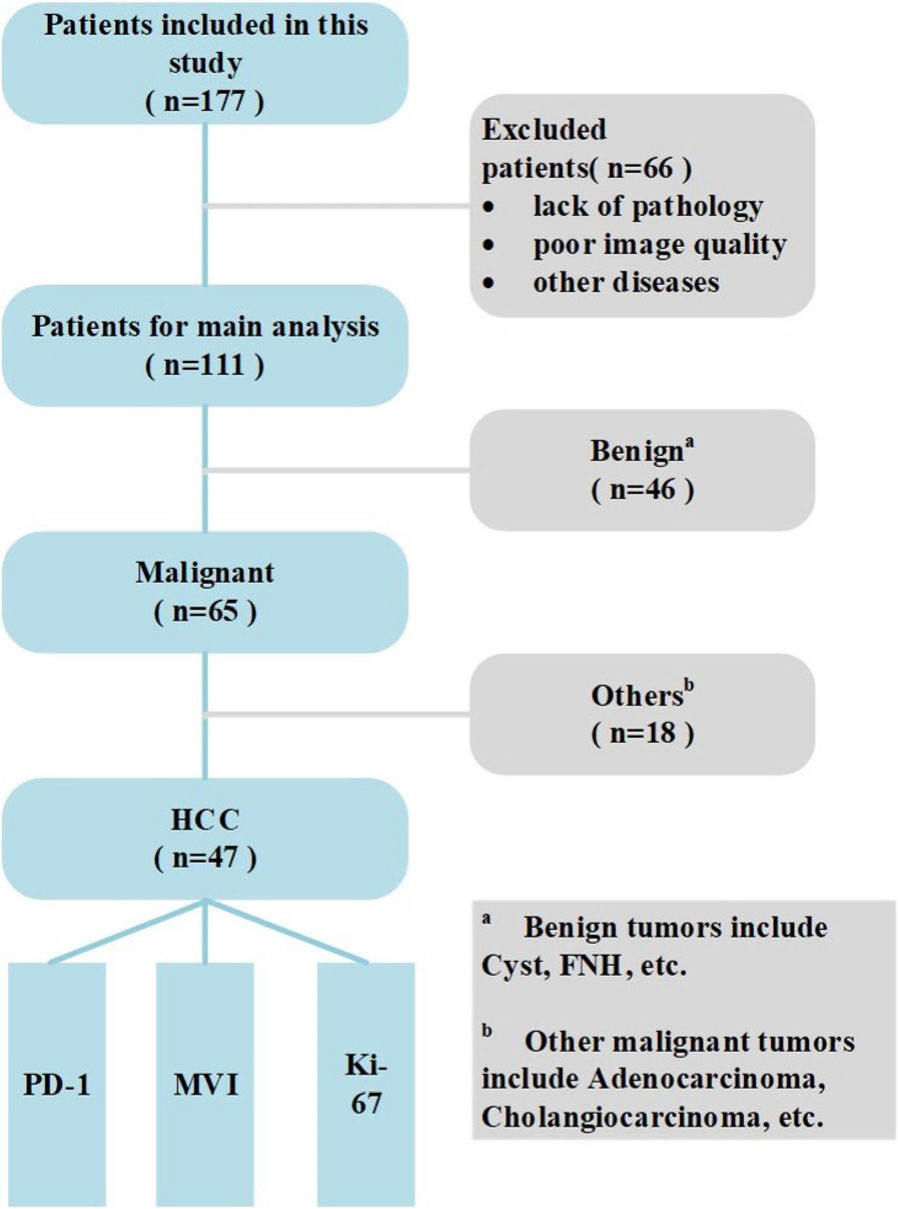
The flowchart of the proposed HCC diagnostic and prediction system

The flow chart of the proposed radiomics system is shown in Fig. 1. We first classified the benign and malignant cases. Then, we separated patients with HCC from the remaining 65 patients with malignancies. Finally, multi-modal ultrasound images of 47 patients with HCC were used to predict PD-1, Ki-67 and MVI indicators of HCC. Benign tumors in the study mainly include cyst and focal nodular hyperplasia (FNH). Other malignant tumors that differ from HCC include adenocarcinoma and cholangiocarcinoma.

Multi-modal ultrasound examinations were performed using Toshiba Aplio i900 ultrasound equipment (Canon Medical, Japan). A PV1-475BX convex array probe (1–8 MHz) was used. Patients lied in a supine position with the right arm in maximal extension. The transducer was positioned in a right intercostal space to visualize the right liver lobe. Large vessels were avoided. Optimally, patients were instructed to perform a transient breath hold in a neutral position. Regions of interest (ROIs) were placed a minimum of 1–2 cm and a maximum of 8 cm beneath the liver capsule [17]. An ROI was placed inside the lesion or surrounding the hepatic parenchyma at the same depth as the lesion. In the ROIs of lesions and the parenchyma, SWE and viscosity were measured.

**Fig. 2:**
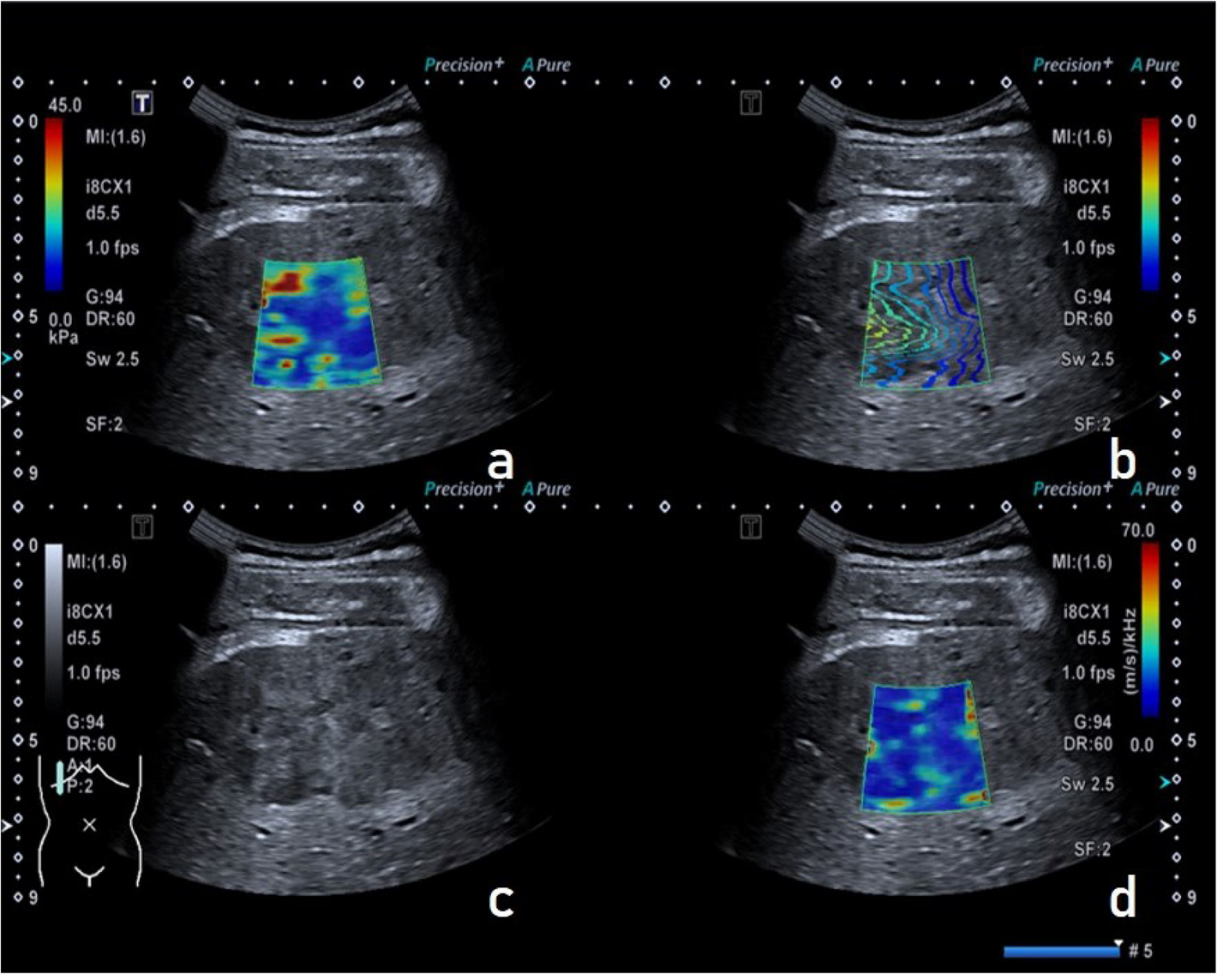
Multi-modal colour ultrasound image. **a**. Elastography. **b**. Propagation map, which reflects the image quality. **c**. Gray-scale ultrasound. **d**. Viscosity modality

A multi-modal ultrasound image includes four different modalities, as shown in Fig. 2, where the upper left panel is an elastography image, the lower left panel is a gray-scale ultrasound image and the lower right panel is a viscosity image. The propagation map in the upper right panel reflects the image quality (> 90% was considered to be good quality for measurement). The regions with optimal and stable imaging qualities were manually selected by an experienced sonographer and marked as ROIs.

### Overall design

The overall methods include three steps: feature extraction, feature selection and classification. First, the SR dictionary was trained to extract features. Then, an iterative algorithm based on SR was used for feature selection. Finally, we trained an SVM model with the selected features. We validated the model by leave-one-out cross-validation (LOOCV).

### Feature extraction

We adopted an SR-based feature extraction method to extract image features. First, we used the K-singular value decomposition (KSVD) algorithm to learn the corresponding structural texture dictionary from each type of image [18]. Then, the various types of dictionaries were combined into a feature extraction dictionary (FED), and the FED was used to sparsely represent the test images. The representation coefficients reflect the relationship between the test images and each type of dictionary (each class), so the coefficients can be classified as the test image features. We used the orthogonal matching pursuit (OMP) algorithm to calculate the SR coefficients and extract the coefficients for features. The detailed process of feature extraction can be found in Appendix: Feature extraction.

### Feature selection

Redundant and irrelevant features can seriously affect the performance of the classification. Hence, we adopted an iterative SR method to select some crucial features for the classifier. We used sample features to sparsely represent sample labels, and the absolute value of the SR coefficient was the importance of the feature. To improve the stability of feature selection, we performed iterative SR for feature selection. We selected a partial sample for SR in each iteration and then averaged the results of multiple SRs to determine the final coefficients. Finally, we sorted the features according to the absolute value of the SR coefficients. Specific mathematical models for feature selection can be found in Appendix: Feature selection.

### Classification

There are many types of classifiers in radiomics, and SVM is widely used for stability and optimal performance. In this work, we used LibSVM for classification, which can solve the problem of sample imbalance [19]. A specific mathematical model of LibSVM is shown in Appendix: SVM model. By adjusting the penalty factor, we eliminated the effects of sample imbalance. A receiver operating characteristic (ROC) curve was used to show the overall performance of the model. We also calculated some indexes to evaluate the performance of the classifier, including accuracy (ACC), sensitivity (SENS), specific (SPEC) and area under the ROC (AUC).

### Cross-validation

Each time LOOCV takes one sample as a test sample, and all the remaining samples are used as training sets. This process was repeated until all the samples were traversed. We used LOOCV to evaluate our model.

### Statistical analysis

Descriptive statistics are summarized as the mean ± SD. The Mann-Whitney U test was used to test whether a feature has discriminative power in different tasks, and *p* values less than 0.05 indicated statistical significance. SPSS statistics 20.0 software (SPSS, Chicago, IL, USA) and MedCalc software (V.11.2; 2011 MedCalc Software bvba, Mariakerke, Belgium) were used to perform the statistical analysis.

## Results

### Multi-modal ultrasound image feature extraction and feature analysis

**Fig. 3:**
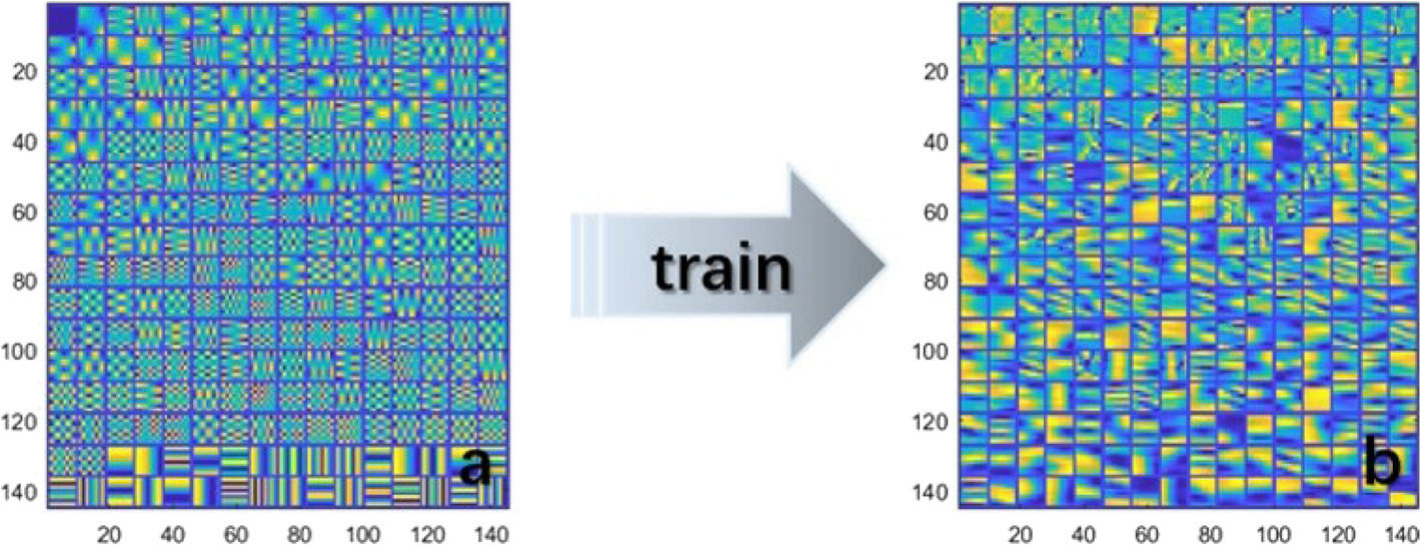
A schematic diagram of dictionary training. **a**. Initial DCT dictionary; **b**. dictionary after training

Because the model establishment process was similar for the five radiomics models, we used benign and malignant differentiation as an example analysis. A schematic diagram of the dictionary training is shown in Fig. 3. Figure 3a shows a blank dictionary that has not been trained. Because the initial discrete cosine transform (DCT) dictionary cannot optimally represent the image information of each category simultaneously, it was necessary to train different dictionaries that include the texture structure features of each type based on the DCT dictionary. We use the KSVD algorithm to train the dictionary, and we finally obtained a dictionary with rich texture information, as shown in Fig. 3b.

**Fig. 4:**
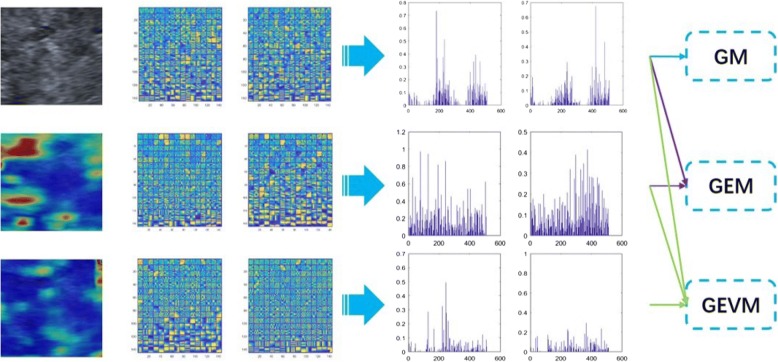
The overall flowchart of feature extraction. Features were extracted from different modal images and then combined. GM represents the gray-scale modality; GEM represents the gray-scale and elastography modality; GEVM represents the gray-scale, elastography and viscosity modality

The overall flowchart of feature extraction is shown in Fig. 4. We manually selected three corresponding square measurement areas as the ROIs in the multi-modal images. The size of the dictionary we used in this study is 64 × 256. A dictionary contains 256 atoms, corresponding to 256 sparse coefficients, which can be taken as 256 features. In the case of using only gray-scale ultrasound images, two dictionaries need to be trained separately for the two categories, so a total of 512 features can be extracted. Particularly, when extracting features from elastography or viscosity modality images, because the image is three channels (RGB), we first performed HSV (hue, saturation, value) conversion on the RGB images. Then, we used the hue (H) and value (V) channels to train the dictionary separately. Hence, for elastography or viscosity images, we trained four dictionaries to obtain 1024 features. Finally, after multi-modal feature combination, the gray-scale modality (GM), gray-scale and elastography modality (GEM) and gray-scale, elastography and viscosity modality (GEVM) corresponded to 512, 1536 and 2560 features, respectively.

**Fig. 5:**
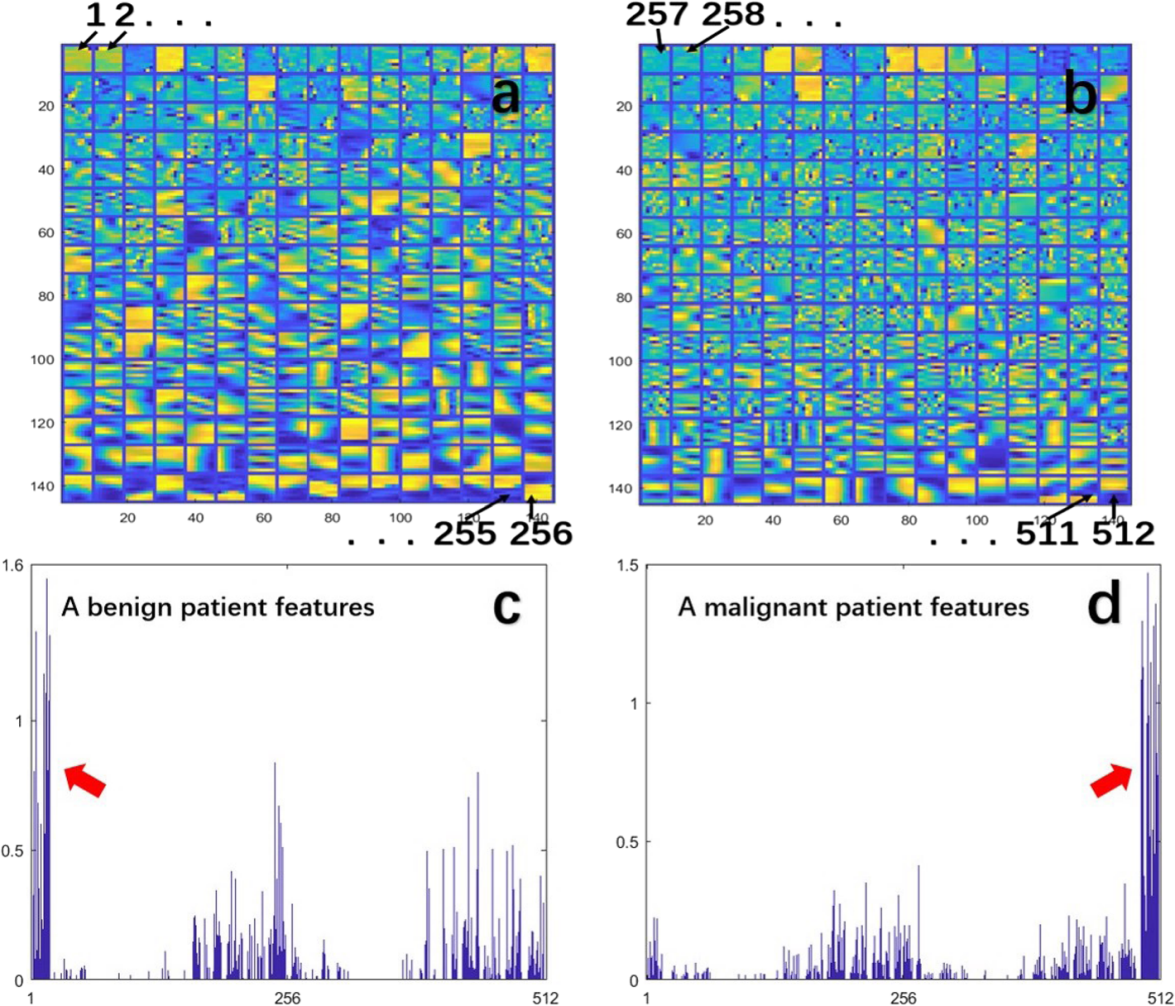
Benign and malignant dictionaries and the feature amplitudes of the two cases. The feature amplitudes of the two cases are concentrated in different areas so that they can be distinguished. **a**. Benign dictionary; **b**. malignant dictionary; **c**. feature amplitude of the benign case; **d**. feature amplitude of the malignant case

We randomly selected two cases (one benign and the other malignant) to analyse the features of their GM images. The feature amplitudes of the two cases and two corresponding benign and malignant dictionaries are shown in Fig. 5. Figure 5a and b correspond to benign and malignant dictionaries, respectively, and they together form an FED. In the two dictionaries, 512 atoms correspond to 512 features of a case. It is obvious that the two dictionaries have quite different textures and that the malignant dictionary has more structural information. The linear combination of atoms in FED makes up the entire ROI, and the different feature magnitudes represent the different proportions of atoms. The special region in Fig. 5 is marked by a red arrow. The area with the highest amplitude of the benign patient is located in the feature interval corresponding to the benign dictionary (1 to 256), while the area with the highest amplitude of the malignant patient is located in the 257 to 512 feature interval, which corresponds to the malignant dictionary. This result indicates that the image of the benign case is mainly composed of textures from the benign dictionary, while the image of the malignant case is mainly composed of textures from the malignant dictionary. This significant difference can distinguish benign and malignant tumors effectively.

### Feature selection results

**Fig. 6:**
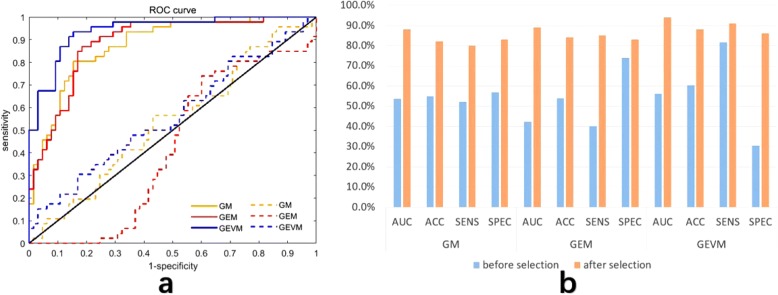
Comparison of benign and malignant classification model performance before (dashed line) and after (solid line) feature selection. **a**. Comparison of the ROC curves of the model. **b**. Histogram comparison of model performance. Both figures show that feature selection has achieved good effects

Eliminating redundant and invalid features is critical to the performance of the classifier. As an example, we analysed the importance of feature selection in benign and malignant tumor classification. Figure 6 shows a comparison of the performance of the features before and after feature selection. Under all imaging modalities, each evaluation indicator of the model has been improved by feature selection. Figure 6a shows a comparison of the ROC curves of the models. The dashed line and solid line correspond to the results before feature selection and after feature selection, respectively. The histogram in Fig. 6b describes the AUC before and after feature selection. The blue bar represents the AUC before feature selection, while the yellow bar corresponds to AUC after feature selection. The results clearly show that our feature selection strategy has achieved good results. The detailed statistical results are shown in Table 2.

**Table 2:** Performance comparison of models before and after feature selection

	GM				GEM				GEVM			
	AUC	ACC	SENS	SPEC	AUC	ACC	SENS	SPEC	AUC	ACC	SENS	SPEC
BF	54	55	52	57	42	54	40	74	56	60	82	30
AF	88	82	80	83	89	84	85	83	94	88	91	86

*AUC* area under the receiver operating characteristic curve, *ACC* accuracy, SENS sensitivity, SPEC specificity, *GM* gray-scale modality, *GEM* gray-scale and shear wave elastography modality, *GEVM* gray-scale, shear wave elastography and viscosity modality, *BF* before selection, *AF* after selection. The auc, acc, sens and spec are expressed as a percentage

### Classification of benign and malignant liver tumors

A total of 111 cases were used in this experiment, of which 65 were malignant cases. We compared the performance of GM, GEM and GEVM in the classification of benign and malignant liver tumors. Some indicators of the model are summarized in Table 3.

**Table 3:** Diagnostic performance of GM,GEM and GEVM for classifying benign and malignant tumors

	AUC(%)	ACC(%)	SENS(%)	SPEC(%)
GM	88	82	80	83
GEM	89	84	85	83
GEVM	94	88	91	86

*AUC* area under the receiver operating characteristic curve, *ACC* accuracy, *SENS* sensitivity, *SPEC* specificity, *GM* gray-scale modality, *GEM* gray-scale and shear wave elastography modality, *GEVM* gray-scale, shear wave elastography and viscosity modality

**Fig. 7:**
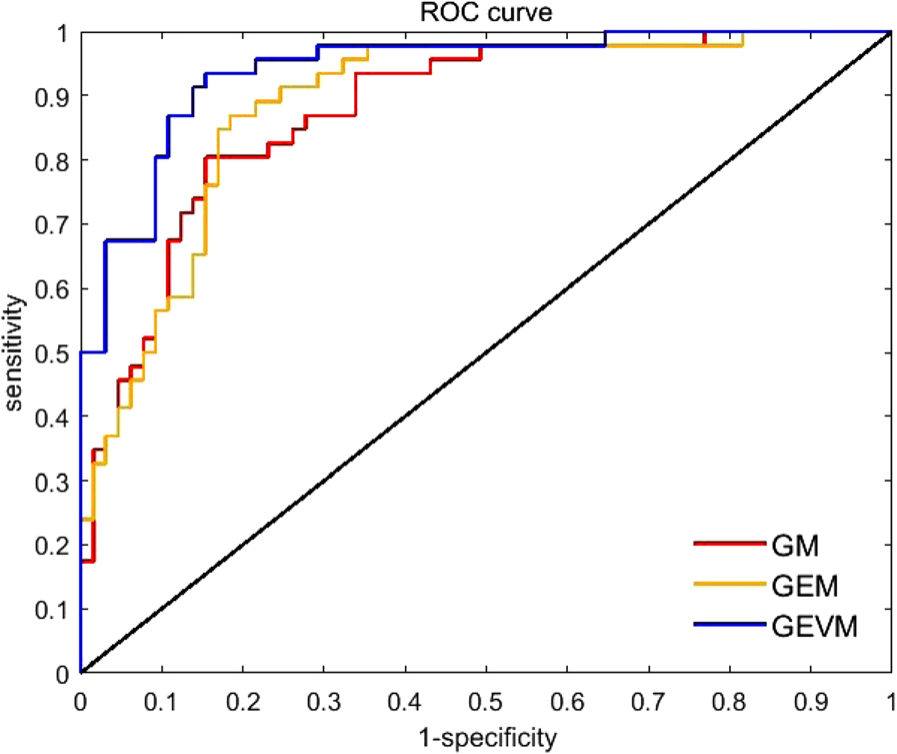
Receiver operating characteristic (ROC) curves of benign and malignant classifications

The AUCs of GEVM and GEM reach 0.94 (95% confidence interval [CI]: 0.88 to 0.98) and 0.89 (CI: 0.81 to 0.94), respectively, which are 0.06 and 0.01 higher than that of GM (CI: 0.80 to 0.93). The AUC of GEVM is 0.05 higher than that of GEM. The ROC curves of these models are shown in Fig. 7. We calculated the statistical significance level of the AUCs for GM and GEVM (*p* = 0.14). Although the application of multi-modal images increased the AUCs, multi-modal images do not exhibit significant differences from single BMUS in terms of differentiation between benign and malignant tumors.

### Malignant liver tumor subcategories

**Fig. 8:**
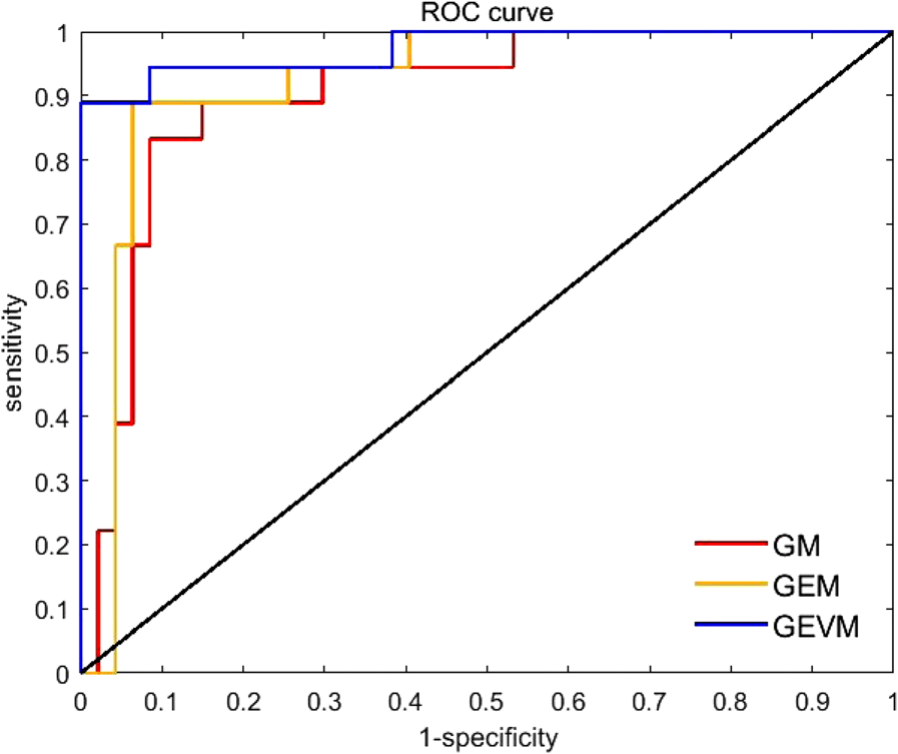
Receiver operating characteristic (ROC) curves of tumor subcategories

A total of 47 HCC and 18 other malignant tumor cases (11 adenocarcinoma cases and 7 cholangiocarcinoma cases) were studied in this experiment. The AUC of GM reached 0.90 (CI: 0.85 to 0.96). The AUCs of GEM and GEVM are slightly greater than that of GM, reaching 0.92 (CI: 0.86 to 0.97) and 0.97 (CI: 0.93 to 0.99), respectively. The ROC curves of these models are shown in Fig. 8. The calculation results show that there are significant differences between GM and GEVM (*p* = 0.04). The application of multi-modal images achieved better results in distinguishing the subtypes of malignant tumors. The results for classification of the subtypes of malignant liver tumors are shown in Table 4.

**Table 4:** Diagnostic performance of GM,GEM and GEVM for liver tumor subtyping

	AUC(%)	ACC(%)	SENS(%)	SPEC(%)
GM	90	89	83	91
GEM	92	92	89	94
GEVM	97	97	89	100

*AUC* area under the receiver operating characteristic curve, *ACC* accuracy, *SENS* sensitivity, *SPEC* specificity, *GM* gray-scale modality, *GEM* gray-scale and shear wave elastography modality, *GEVM* gray-scale, shear wave elastography and viscosity modality

### PD-1, Ki-67, and MVI indicator prediction

**Fig. 9:**
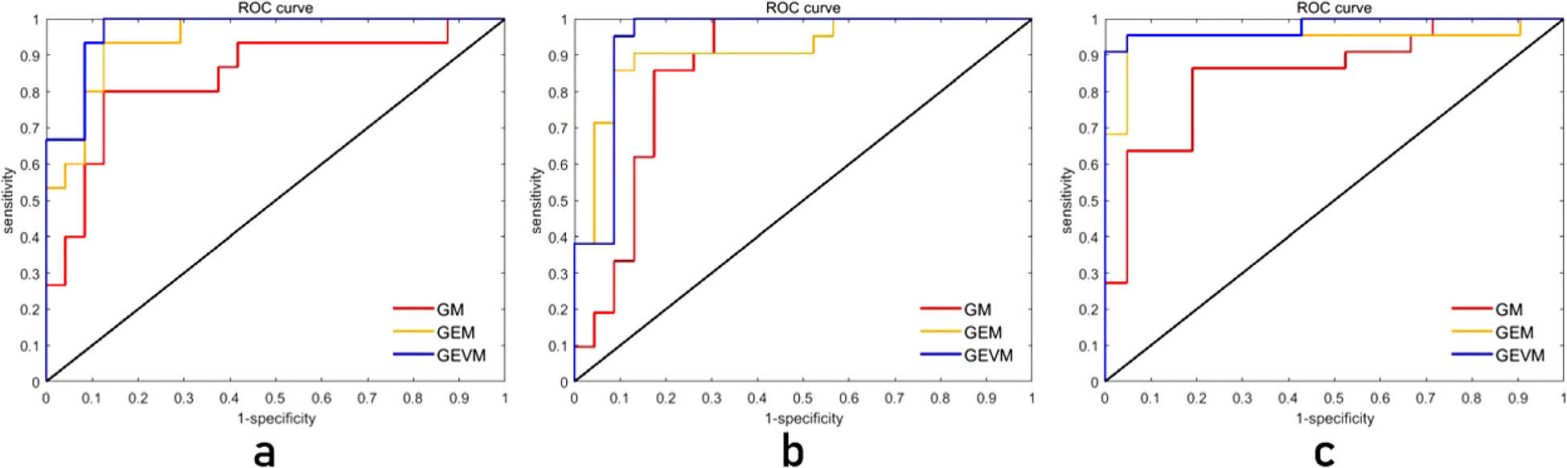
Receiver operating characteristic (ROC) curves of indicator prediction. **a**. ROC curve of PD-1 prediction. **b**. ROC curve of Ki-67 prediction. **c**. ROC curve of MVI prediction

The classification criterion of PD-1 is whether or not the indicator is expressed. The Ki-67 indicator is classified by a 25% threshold value (≤25% or > 25%). The MVI indicator is divided into two categories according to low risk and high risk. The prediction results of the three indicators are summarized in Table 5. The ROC curves of each indicator are shown in Fig. 9. GEVM resulted in significant differences in the AUCs of the three predictive indicators (*p* = 0.02 for PD-1, p = 0.04 for Ki-67, *p* = 0.0006 for MVI) relative to those of GM. Better performance can be obtained by predicting three indicators using multi-modal ultrasound images.

**Table 5:** Performance of GM,GEM and GEVM for indicators prediction

	PD-1				Ki-67				MVI			
	AUC	ACC	SENS	SPEC	AUC	ACC	SENS	SPEC	AUC	ACC	SENS	SPEC
GM	84	85	80	88	86	84	86	83	85	84	86	81
GEM	94	90	93	88	92	89	86	91	95	93	91	95
GEVM	97	92	100	88	94	93	95	91	98	95	91	100

*AUC* area under the receiver operating characteristic curve, *ACC* accuracy, *SENS* sensitivity, *SPEC* specificity, *GM* gray-scale modality, *GEM* gray-scale and shear wave elastography modality, *GEVM* gray-scale, shear wave elastography and viscosity modality, *PD-1* programmed cell death protein 1, *Ki-67* antigen Ki 67, *MVI* micro vascular invasion. The auc, acc, sens and spec are expressed as a percentage

## Discussion

Multi-modal ultrasound technology increases the diagnostic efficiency of ultrasound and makes it possible to diagnose FLL before surgery. In contrast to the evaluation of diffuse parenchymal liver disease, little is known about FLL characterization using SWE or SWV technology. Here, we investigate the value of multi-modal ultrasound technology for the differential diagnosis of benign and malignant FLLs using radiomics analysis. Previously, Dong et al. [20] applied ElastPQ measurements for differential diagnosis of benign and malignant FLLs and successfully found the optimal threshold of shear wave speed. Ozmen et al. [21] used the optimal threshold of SWE to differentiate benign and malignant liver tumors and obtained an AUC of 0.77. However, the cut-off values of measurement for differentiating benign and malignant liver tumors tend to show great variability. In our study, innovative multi-modal ultrasound images were used to diagnose liver tumors. By converting the images into high-throughput features, radiomics was used to mine the rich texture information in the patient images in order to classify the images. We found that malignant tumor images have more complex textures and more structural information. The experimental results also show that the model has achieved good results on the classification of benign and malignant liver tumors (0.94 AUC for differentiating between benign and malignant liver tumors).

The most common type of histology of primary liver cancer is HCC, which represents 90% of cases [22, 23]. Difficulties in treatment and poor prognosis make it important to accurately detect HCC. In addition, early diagnosis of HCC is also crucial for optimizing treatment options. In a study by Thomas et al., alpha-fetoprotein (AFP) was used to detect HCC [24]. However, AFP is only a supplement to the ultrasound image information, and the accuracy of detecting HCC is not satisfactory. In our experiments, multi-modal ultrasound images were used to directly distinguish between HCC and other malignancies noninvasively, and the model performed well (0.97 AUC for liver tumor subtyping). This result illustrates the great potential of ultrasound images for tumor diagnosis.

Patients with HCC have a poor prognosis due to a high recurrence rate. It has been reported that the 5-year recurrence rate of primary liver cancer is as high as 45%~ 60% [25]. We mainly studied two factors that affect the recurrence of liver cancer. One of the factors is MVI. MVI has been reported as one of the major risk factors related to HCC recurrence and represents a poor prognosis [26, 27]. Many previous studies have focused on identifying radiologic features (such as tumor size, tumor margin, and number of lesions) in various types of medical images for the preoperative prediction of MVI [28–30]. However, the best predictive feature of MVI in HCC remains controversial. In addition, another study used a radiomics nomogram to predict MVI preoperatively, resulting in a C-index of 0.84 [31]. However, the results of these studies are not satisfactory. Our radiomics-based model achieved better results (0.98 AUC) in predicting MVI than did previous studies using multi-modal ultrasound images.

Another factor we studied that has an effect on HCC recurrence is Ki-67. A previous study suggested that a higher Ki-67 index confers poor prognosis in patients with HCC [32–34]. Clinically, immunohistochemistry is needed to detect the Ki-67 index. Studies have analysed the correlation between the expression of other proteins (such as PDIA3) and Ki-67 [35]. However, to the best of our knowledge, no study has applied medical images to predict Ki-67 noninvasively. Our results (0.94 AUC for Ki-67 prediction) demonstrated that it is feasible to noninvasively predict Ki-67 based on radiomics. In our study, we successfully determined MVI and Ki-67 for HCC prognosis by applying multi-modal ultrasound images.

Recent studies have shown that immunotherapy is a promising approach for HCC treatment and that PD-1 is crucial for tumor immunity [36]. Accurate assessment of PD-1 can be useful in assessing the range of applications of PD-1/PD-L1 blockers in liver cancer patients. In addition, an increase in PD-1 predicts a poorer prognosis for HCC [37]. The prediction of PD-1 is important for the progression and postoperative recurrence of HCC. The model we built for PD-1 prediction has achieved good results (0.97 AUC for PD-1 prediction). By integrating multi-modal ultrasound image information, the radiomics model can determine PD-1 noninvasively.

To investigate the effects of feature selection on classifier performance, we compared the performance of models before and after feature selection in benign and malignant tumors. Feature selection truncates redundant and invalid features, so the model becomes robust. The experimental results show that the performance of the model after feature selection is better than that before feature selection (significant level in ROC curves, *p* < 0.0001).

There are some limitations to our research. It should be mentioned that our study lacks multi-centre validation, which would provide more convincing results. In addition, more samples should be collected to build a more robust model. Furthermore, we employed only the image information from diseased livers, and some text descriptions of the cases and biomarkers were not applied.

## Conclusions

In summary, we successfully established an HCC diagnosis and prognosis system based on ultrasound radiomics and proved its potential feasibility and effectiveness. Simultaneously, we demonstrated the potential value of multi-modal ultrasound-based radiomics analysis in computer-aided diagnosis (CAD).

## Appendix

### Feature extraction

The SR method can adaptively learn and extract texture features of images. First, we exploited the KSVD algorithm to train the dictionary corresponding to different categories. This algorithm trains different categories of dictionaries by iteratively updating each atom in the dictionary. We denote **i ∈** {**1**, **2**, …, ***I***} as all sample categories. ***D***_***i***_ is the corresponding i-class dictionary. Then, the process of feature extraction can be written as:

1 $$ \widehat{\boldsymbol{\alpha}}=\mathbf{\arg}{\mathbf{\min}}_{\boldsymbol{\alpha}}{\left\Vert \boldsymbol{y}-\boldsymbol{D}\boldsymbol{\alpha } \right\Vert}_{\mathbf{2}}^{\mathbf{2}}+\boldsymbol{\mu} {\left\Vert \boldsymbol{\alpha} \right\Vert}_{\boldsymbol{p}} $$

where **y** is classifier label; **D** = [***D***_**1**_, ***D***_**2**_, ⋯, ***D***_***I***_] is a collection of all SR dictionaries; **α** is the SR coefficients, which can be considered as features of the samples; $\widehat{\boldsymbol{\alpha}}$ is the estimated value of **α**; ‖∙‖_***p***_ represents the ***l***_***p***_ norm; ***μ*** is the regularization parameter. ***μ***‖***α***‖_***p***_ can be regarded as the error term that can be discarded. We used the OMP algorithm to solve (1) to obtain the image features.

### Feature selection

Different from the traditional feature selection method, the SR method adopts the strategy of the sliding window, so it can comprehensively utilize the information of all samples in the window. An iterative process can be expressed as:

2 $$ {\widehat{\boldsymbol{d}}}^{\left(\boldsymbol{k}\right)}={\mathbf{argmin}}_{\boldsymbol{d}}{\left\Vert {\boldsymbol{s}}^{\left(\boldsymbol{k}\right)}-{\boldsymbol{F}}^{\left(\boldsymbol{k}\right)}\boldsymbol{d}\right\Vert}_{\mathbf{2}}^{\mathbf{2}}+\boldsymbol{\varepsilon} {\left\Vert \boldsymbol{d}\right\Vert}_{\mathbf{0}} $$

where ***s***^(***k***)^ is the label used for the **k**-th iteration; ***F***^(***k***)^ is the feature selected for the **k**-th iteration; ***ε*** is a small constant; ${\widehat{\boldsymbol{d}}}^{\left(\boldsymbol{k}\right)}$ is the coefficient calculated by the **k**-th iteration. Then, we calculated the average of ${\widehat{\boldsymbol{d}}}^{\left(\boldsymbol{k}\right)}$ for the **k**-th iteration:

3 $$ {\boldsymbol{d}}^{\left(\boldsymbol{k}\right)}=\frac{\mathbf{1}}{\boldsymbol{k}}\sum \limits_{\boldsymbol{i}=\mathbf{1}}^{\boldsymbol{k}}{\widehat{\boldsymbol{d}}}^{\left(\boldsymbol{k}\right)} $$

The ***d***^(***k***)^ was used for feature selection. After the iteration, each feature obtained a score that combines all the sample information due to the averaging operation. The higher the score, the higher the importance of the feature. In this way, the feature selection results are obtained.

### SVM model

The LibSVM model can solve the sample imbalance problem by adjusting different penalty coefficients. The improved SVM mathematical model can be written as:

4 $$ {\mathbf{\min}}_{\boldsymbol{w},\boldsymbol{b},\boldsymbol{\xi}}\kern1em \frac{\mathbf{1}}{\mathbf{2}}{\boldsymbol{\omega}}^{\boldsymbol{T}}\boldsymbol{\omega} +{\boldsymbol{C}}_{+}{\sum}_{{\boldsymbol{y}}_{\boldsymbol{i}=\mathbf{1}}}{\boldsymbol{\xi}}_{\boldsymbol{i}}+{\boldsymbol{C}}_{-}{\sum}_{{\boldsymbol{y}}_{\boldsymbol{i}=-\mathbf{1}}}{\boldsymbol{\xi}}_{\boldsymbol{i}} $$

5 $$ \mathbf{subject}\ \mathbf{to}\kern0.75em {\boldsymbol{y}}_{\boldsymbol{i}}\left({\boldsymbol{\omega}}^{\boldsymbol{T}}\boldsymbol{\phi} \left({\boldsymbol{x}}_{\boldsymbol{i}}\right)+\boldsymbol{b}\right)\mathbf{\ge}\mathbf{1}-{\boldsymbol{\xi}}_{\boldsymbol{i}} $$

$$ {\boldsymbol{\xi}}_{\boldsymbol{i}}\mathbf{\ge}\mathbf{0},\boldsymbol{i}=\mathbf{1},\dots, \boldsymbol{l} $$

where ***ω*** is the hyperplane normal vector and ***b*** is the bias, which collaboratively determines the hyperplane; ***ϕ***(***x***_***i***_) is the feature vector mapped by ***x***_***i***_; ***y***_***i***_ is the sample label; ***ξ***_***i***_ is a small constant; ***C***_***+***(***−***)_ is the penalty parameter, which assigns weights to different proportions of samples. By assigning an appropriate ***C***, we can eliminate the sample imbalance problem.

## Abbreviations

ACC: accuracy
AFP: alpha-fetoprotein
AUC: area under the receiver operating characteristic curve
BMUS: B-mode ultrasound
CAD: computer-aided diagnosis
CI: confidence interval
DCT: discrete cosine transform
FED: feature extraction dictionary
FLLs: focal liver lesions
FNH: focal nodular hyperplasia
GEM: gray-scale and elastography modality
GEVM: gray-scale, elastography and viscosity modality
GM: gray-scale modality
HCC: hepatocellular carcinoma
Ki-67: antigen Ki-67
KSVD: K-singular value decomposition
LOOCV: leave-one-out cross-validation
MVI: microvascular invasion
OMP: orthogonal matching pursuit
PD-1: programmed cell death protein 1
ROC: receiver operating characteristic
SENS: sensitivity
SPEC: specificity
SR: sparse representation
SRT: sparse representation theory
SVM: support vector machine
SWE: shear wave elastography
SWV: shear wave viscosity
